# RCSB Protein Data Bank (PDB): Driving Research and Education Using Experimentally-Determined Structures and Computed Structure Models from Artificial Intelligence/Machine Learning

**DOI:** 10.1063/4.0000914

**Published:** 2025-10-27

**Authors:** Yuhe Liang, Christine Zardecki, RCSB PDB Team

**Affiliations:** RCSB Protein Data Bank (PDB), Piscataway, NJ, USA

## Abstract

The 2024 Nobel Prize in Chemistry was awarded to David Baker, Demis Hassabis, and John Jumper for computational protein design and protein structure prediction. These remarkable achievements depended critically on open access to the atomic-level, experimentally- determined, three-dimensional (3D) structures of macromolecules archived in the Protein Data Bank (PDB).

RCSB.org is the research-focused web portal of the RCSB Protein Data Bank (RCSB PDB) that provides open access >230,000 PDB structures alongside >1 million Computed Structure Models (CSMs) generated using AlphaFold2 (from AlphaFoldDB), and RoseTTAFold and AlphaFold2 (from ModelArchive). For the avoidance of doubt, experimentally-determined PDB structures and CSMs delivered on RCSB.org by the RCSB PDB are clearly identified as to their respective provenance and reliability.

RCSB.org users can query, organize, visualize, analyze, and compare experimental PDB structures and CSMs side-by-side by utilizing powerful tools:

Search: User queries can be applied to all PDB structures *and* CSMs; PDB structures only; and can exclude either PDB structures *or* CSMs from the search results. N.B.: To reduce the likelihood of confusion with experimentally-determined structures, CSMs are not included in the search unless the RCSB.org user “opts in.”.

*View and Organize Results*: By default, search results are ordered based on a relevancy score. Results can be resorted using various criteria (e.g., listing experimental PDB structures first, global per-residue confidence score (pLDDT)).

*Explore Similar Proteins*: "Group" summary pages and search results simplify exploration of PDB structures with the same UniProt ID or sequence similarity or were deposited as part of the same study.

*Explore Individual Structures*: Structure Summary Pages offer details of either experimental PDB structures and CSMs.

Assess quality: Analogous to the validation slider for experimental structures, all CSMs report global and local confidence levels as pLDDT scores.

Visualize in 3D: View experimental PDB structures and CSMs in Mol*. Use the standalone Mol* 3D Viewer to upload single or multiple data files, align structures, and run Structure Motif Search.

*Download*: From Structure Summary Pages, download atomic coordinates for the PDB structure (various formats) or those of a CSM in ModelCIF format hosted by the corresponding external archive.

RCSB PDB will continue to develop resources to support exploration of experimentally-determined PDB structures alongside CSMs, scaling infrastructure and operations to accommodate growth in the PDB archive and increased availability of predicted structures.

RCSB PDB offers a variety of resources to support graduate students, postdoctoral fellows, and researchers in CSM exploration, including virtual and in-person events, with related materials published at PDB-101 (PDB101.RCSB.org) and user guidance documentation published at RCSB.org.

PDB-101 hosts an additional collection of materials focused on protein prediction and protein design.

RCSB PDB Core Operations are funded by National Science Foundation (DBI-2321666), US Department of Energy (DE-SC0019749), and National Cancer Institute, National Institute of Allergy and Infectious Diseases, and National Institute of General Medical Sciences of the National Institutes of Health under grant R01GM157729.

